# Supplementary material for: Asynchronous magnetic resonance elastography: Shear wave speed reconstruction using noise correlation of incoherent waves
Source: Magn Reson Med. 2022 Oct 27;89(3):990–1001. doi: 10.1002/mrm.29502 (PMC9792433; doi:10.1002/mrm.29502)
Supplement: Supplementary file 1 — DATA S1 MATLAB code used to generate the numerical simulations of Figure 1 [file MRM-89-990-s002.zip › k-Wave/helpfiles/unmaskSensorData.html]

unmaskSensorData :: Functions (k-Wave)


# unmaskSensorData

Reorder data recorded using a binary sensor mask.

## Syntax

```
unmasked_sensor_data = unmaskSensorData(kgrid, sensor, sensor_data)
```

## Description

`unmaskSensorData` restores the grid position of the data recorded at a single time-step within the time-series data returned by `kspaceFirstOrder1D`, `kspaceFirstOrder2D`, or `kspaceFirstOrder3D` when using a binary sensor mask.

## Inputs

|  |  |
| --- | --- |
| `kgrid` | k-Wave grid object returned by `kWaveGrid` |
| `sensor` | k-Wave sensor structure where `sensor.mask` is defined as a binary grid |
| `sensor_data` | sensor data (returned by the first order simulation functions) at a single time-step ordered using MATLAB's standard column-wise linear matrix indexing |

## Outputs

|  |  |
| --- | --- |
| `unmasked_sensor_data` | matrix with the sensor data reordered to its original position on the sensor mask |

## See Also

`kWaveGrid`, `kspaceFirstOrder1D`, `kspaceFirstOrder2D`, `kspaceFirstOrder3D`, `reorderSensorData`
